# Extraordinary behavioral entrainment following circadian rhythm bifurcation in mice

**DOI:** 10.1038/srep38479

**Published:** 2016-12-08

**Authors:** Elizabeth M. Harrison, Thijs J. Walbeek, Jonathan Sun, Jeremy Johnson, Qays Poonawala, Michael R. Gorman

**Affiliations:** 1 Center for Circadian Biology, University of San Diego, California, 9500 Gilman Dr. La Jolla, CA 92093, USA; 2 Department of Psychology, University of California, San Diego, 9500 Gilman Dr. La Jolla, CA 92093, USA

## Abstract

The mammalian circadian timing system uses light to synchronize endogenously generated rhythms with the environmental day. Entrainment to schedules that deviate significantly from 24 h (T24) has been viewed as unlikely because the circadian pacemaker appears capable only of small, incremental responses to brief light exposures. Challenging this view, we demonstrate that simple manipulations of light alone induce extreme plasticity in the circadian system of mice. Firstly, exposure to dim nocturnal illumination (<0.1 lux), rather than completely dark nights, permits expression of an altered circadian waveform wherein mice in light/dark/light/dark (LDLD) cycles “bifurcate” their rhythms into two rest and activity intervals per 24 h. Secondly, this bifurcated state enables mice to adopt stable activity rhythms under 15 or 30 h days (LDLD T15/T30), well beyond conventional limits of entrainment. Continuation of dim light is unnecessary for T15/30 behavioral entrainment following bifurcation. Finally, neither dim light alone nor a shortened night is sufficient for the extraordinary entrainment observed under bifurcation. Thus, we demonstrate in a non-pharmacological, non-genetic manipulation that the circadian system is far more flexible than previously thought. These findings challenge the current conception of entrainment and its underlying principles, and reveal new potential targets for circadian interventions.

The circadian timing system in mammals is coordinated by the light-responsive suprachiasmatic nuclei in the anterior hypothalamus. While it has been long established that bright light adaptively synchronizes the pacemaker to the solar day, irradiances equivalent in intensity to moonlight and starlight also exert powerful effects on the circadian system. This dim scotophase illumination (DSI), in comparison with completely dark nights, markedly enhances elasticity of circadian entrainment across multiple experimental protocols. Specifically, DSI reduces re-entrainment time to simulated timezone travel by 50%^1^,^2^; extends the upper range of entrainment to non-24 h days by ~4 hours^3^; and in response to short daylengths, accelerates adoption of the season-typical circadian waveform featuring elongated subjective nights and truncated subjective days^4^.

Of particular relevance, pairing of DSI with exposure to a light/dark/light/dark (LDLD) schedule temporally reorganizes circadian clocks of rodents to produce two periods of alternating activity and rest per 24 hours - an alternative circadian waveform termed “bifurcation.” The daily alternation between biological night and day is thus bifurcated, and the animal experiences two subjective days and nights per 24 h period. Extensive behavioral studies indicate that this reflects a bona fide entrainment state: bifurcation can be maintained under skeleton photoperiods^5^, and non-behavioral markers of subjective day and night – elevated melatonin concentrations, light responsiveness and SCN function – are expressed bimodally in bifurcated animals^5^,^6^,^7^,^8^. The bifurcated entrainment state is maintained by the actions of the twice daily photophases that counteract coupling interactions favoring the unbifurcated state, as the two separate activity bouts will join into a single conventional nighttime activity pattern within a few days in constant conditions^5^,^9^.

While rest/activity intervals in bifurcated animals may repeat every 12 h under some LDLD conditions, this pattern reflects two functionally distinct 24 h oscillations that are roughly in anti-phase rather than a single 12 h oscillation. Indeed, bifurcated rhythms are readily induced and maintained in LDLD cycles where the two nights are not in anti-phase (e.g., LDLD9:5:5:5)^10^. Moreover, even when entrained to symmetrical LDLD cycles (e.g., LDLD7:5:7:5), the phase of activity onsets relative to one scotophase may differ from that with respect to the other scotophase, as does the amount of activity in each bout. Finally, when released into DD, the kinetics of rejoining differ depending on which scotophase begins the transition to constant conditions, indicating the underlying oscillators exert differential effects on one another (i.e., have different coupling mechanisms)^9^. Mechanistically, bifurcation in LDLD is associated with 24 h oscillations of clock gene products that are expressed in anti-phase in core versus shell regions of the SCN^6^,^7^.

A non-intuitive, but translationally attractive, consequence of circadian waveform manipulation is a marked change in the ability of bright light to reset pacemaker phase. Adaptation of hamsters to short versus long photoperiods, for example, increases the amplitude of the light pulse phase response curve (PRC) and reduces forty-fold the sensitivity threshold for photic resetting and induction of SCN early immediate gene expression^11^,^12^,^13^,^14^. Moreover, in response to simulated time-zone travel, short day adaptation reduces a measure of jet-lag by 49% and bifurcation reduces it by 71% compared to long-day entrained hamsters^15^. The evidence of altered resetting plasticity observed after both manipulation of circadian waveform and exposure to DSI prompted us to examine the joint and separate effects of DSI and bifurcation on the ability of animals to entrain to non-24 h days (T cycles) generally considered beyond the range of conventional entrainment. Here we test in three experiments: (1) whether dim light and bifurcation enable entrainment to an LDLD T30 cycle; (2) once bifurcated, whether dim light is necessary for this entrainment; and (3) whether dim light or shot photoperiods on their own are sufficient. Here we demonstrate that DSI, by virtue of its ability to induce bifurcation in mice exposed to 24 h LDLD cycles, enables apparent behavioral entrainment to 30 h LDLD conditions. Following bifurcation, DSI is not necessary for T30 adaptation nor is it sufficient under non-bifurcating conditions.

## Methods and Materials

### General Methods

All procedures were approved by and carried out in accordance with guidelines and regulations of the Institutional Animal Care and Use Committee, University of California, San Diego. Male C57Bl/6J mice aged 4–5 weeks (Jackson Labs; Sacramento, CA) were housed at 22 ± 2 °C with food (Purina Rodent Chow No. 5001, St. Louis, MO) and water provided ad libitum for the entirety of the study. After acclimation to the laboratory, animals were housed singly in polypropylene cages (17.8 × 25.4 × 15.2 cm) to accommodate metal running wheels (11.4 cm diam). Locomotor activity rhythms were monitored with a Vitalview data collection system (Version 4.2, Minimitter, Bend OR) that compiled in 6 min bins the number of electrical closures triggered by half wheel rotations. Cage changes for all singly-housed mice were scheduled at 3-week intervals during a photophase in an effort to minimize disruptions to the spontaneous wheel-running rhythms.

For each experiment, animals were first exposed for 4 weeks to a 24 h light:dark (LD) or light:dark:light:dark (LDLD) baseline photoperiod (Phase 1) followed by an additional 4 week period in a 30 h LD or LDLD cycle (Phase 2). For animals in LDLD (i.e., two light and dark phases per cycle) the cycles may be considered equivalent to 12 h and 15 h LD (i.e, LD7:5 and LD10:5). Because prior work described above demonstrates a 24 rather than 12 h oscillatory basis to rhythm bifurcation in LDLD7:5:7:5, we denote this as a T24 condition. Although rhythmicity under LDLD10:5:10:5 has yet to be characterized, we analogously refer to this condition as T30. Animals were always introduced to T30 cycles by extending the duration of the photophase and keeping the length of the scotophase constant. Under all conditions, photophase illumination was generated by white tube fluorescent lamps at an intensity of 30–100 lux inside individual cages. Scotopic conditions differed by experimental groups: some groups were maintained in complete darkness; others were provided with DSI generated by green LEDs (peak and half max bandwidth = 560 and 23 nm, respectively) at an intensity of <0.1 lux. In Experiments 1 and 3, animals were released into constant conditions following Phase 2 (described below - Phase 3). Animals were humanely euthanized following the final environmental manipulation described for each experiment.

## Experimental Procedure

### Experiment 1

#### Does bifurcation with DSI facilitate behavioral entrainment to T30?

Mice (n = 30) were randomly assigned to one of two groups that differed only in presence or absence of dim illumination during scotophases. In Phase 1, both groups were exposed to alternating 7 h photophases and 5 h scotophases for 4 weeks (24 h LDLD). In Phase 2, mice experienced alternating 10 h photophases and 5 h scotophases (30 h LDLD). Consequently the two groups are designated as LD^im^7:5/LD^im^10:5 (n = 10) and LD^ark^7:5/LD^ark^10:5 (n = 20), respectively. After 4 weeks in Phase 2 conditions, all animals were released into constant darkness (DD; i.e., no dim) for 10 days to allow assessment of free-running period (Phase 3) and analysis of the phase of activity onsets. Tight clustering of onset phases would indicate phase control, while phase dispersion would suggest that rhythms were un-entrained in T30. To assess whether any clustering of phases might occur on a 15 h or 30 h period, half of the animals from each group experienced one additional cycle of LD10:5 prior to constant conditions. Thus, animals were released into DD beginning at one of two successive scotophases (DD_1_ and DD_2_) that were 15 h apart.

### Experiment 2

#### Is DSI necessary for behavioral entrainment to T30 after bifurcation is induced?

Male mice (n = 32) were exposed to 24 h LDLD cycles with dim illumination to promote bifurcation for 4 weeks (Phase 1): The first two weeks were in LD^im^8:4 and the second two weeks in LD^im^7:5, keeping the time of lights off constant. In Phase 2, all mice were exposed to LD10:5 light cycles: half of the mice continued to receive DSI (LD^im^7:5/LD^im^10:5; n = 15), whereas the remaining mice received completely dark scotophases (LD^im^7:5/LD^ark^10:5; n = 16). For all subjects, fourteen days into Phase 2 a power failure occurred that effectively extended one of the scotophases to 11 h. One mouse died prior to assignment to Phase 2.

### Experiment 3

#### Is bifurcation per se necessary for behavioral entrainment to T30 or are DSI and short nights sufficient?

Mice were randomly assigned to one of five groups: A replicate control group was first bifurcated in LD^im^7:5 and subsequently transferred to T30 as described above (LD^im^7:5/LD^im^10:5; n = 8). Two additional groups were exposed to standard (LD14:10) photoperiods with or without DSI in Phase 1 and subsequently assessed for entrainment to T30 non-bifurcating cycles in Phase 2 (LD^im^14:10/LD^im^20:10; n = 16; LD^ark^14:10/LD^ark^20:10; n = 16). To assess whether a single five-hour scotophase per cycle (rather than the two, 5 h scotophases per cycle in LDLD groups in Experiments 1 and 2) permitted entrainment to T30, a fourth group was exposed to only one, short scotophase in both T24 and subsequent T30 conditions (LD^im^19:5/LD^im^25:5; n = 8). To test the hypothesis that T30 yields more symmetric entrainment in LDLD than does T24, a fifth group was maintained in LD^im^7:5 throughout the entire protocol (LD^im^7:5/LD^im^7:5; n = 8) for contrast with the replicate group in LD^im^7:5/LD^im^10:5.

After 4 weeks, all animals were released into DD for two weeks. As in Experiment 1, half of the animals from each bifurcated group were released into DD at the start of each of the two scotophases (DD_1_, DD_2_), 12 or 15 h apart. For long day groups released from T30, half of the animals in each group were released at the start of the scheduled scotophase and half were released 15 h apart during one of the photophases.

### Data analysis

#### Assessment of Behavioral Entrainment

To assess the periodicity of rhythms at specific cycle lengths, or Ts, Lomb-Scargle periodograms were calculated using the last fourteen cycles (24 or 30 h) of activity data for both Phase 1 and Phase 2^16^,^17^. For T24 and T30 LDLD conditions, the periodogram power at 12 and 24 h and/or at 15 and 30 h, respectively, was extracted for each animal. For LD groups, Phase 1 (T24) and Phase 2 (T30) periodogram power was determined at 24 and 30 h, respectively. Additionally, for assessment of any endogenous, un-entrained or free-running activity components in T30, we extracted the maximum value of the periodogram power between 23 and 26 h. Free-running components outside this range were never apparent. Over the same 14-cycle intervals, the percentage of activity occurring in the photophases was also calculated.

#### Bifurcation Symmetry Index

To assess the degree to which activity was evenly divided between successive scotophases, the total number of activity counts in each scotophase (Scoto1, Scoto2) and the daily total activity over every 24 or 30 h interval was determined for each animal in all LDLD groups for both Phase 1 and Phase 2. The percentage of daily activity in the least-active scotophase was doubled to provide an objective measure of bifurcation ranging from 0 (no activity in 1 scotophase) to 1 (activity perfectly and equally divided between scotophases). Thus, the Bifurcation Symmetry Index is the 14 cycle average of the following: min(Scoto1, Scoto2)*2/(Total LDLD activity). If activity is perfectly bifurcated between successive scotophases, the BSI = 1. Any differences between the two scotophases and any daytime activity, including positive phase angles, will lower this index. If activity is concentrated in only one scotophase per 24 or 30 h, the BSI approaches 0.

#### Entrainment Quotient in T30

As a single, summary measure of quality of entrainment in T30, the peak periodogram power in the entrainment range (i.e 15 h for LD10:5 and 30 h for LD20:10 and LD25:5) was divided by the sum of the peak power in the entrainment range and the maximum value of the periodogram power in the circadian range (i.e. 23–26 h). The more activity is concentrated in the circadian, rather than the entrainment range of the periodogram, the lower the Entrainment Quotient will be. We treated this as an ordinal variable, and non-parametric statistics were applied. To estimate predictability of entrainment to LD10:5 based on prior entrainment under LD7:5, the Spearman correlation between the bifurcation symmetry index and EQT30 was calculated for all animals that went from LD7:5 to LD10:5 across all three experiments.

#### DD data

Under constant conditions, free-running period was determined from least-squares regression lines to eye-fit activity onsets in ClockLab (Actimetrics; Wilmette, IL). All conclusions about free-running period in DD were corroborated with periodogram analysis. From eye-fit activity onsets, the projected phase of activity onset on the day of transfer to DD was estimated and analyzed as a function of entrainment history and the time of exposure to DD. To allow for transients, onsets during the first 72 h in DD were disregarded, and estimates were based on the 7 subsequent values.

For animals with a subjectively apparent free-running rhythm in the circadian range in T30, activity onsets for the free-running component were additionally determined over the seven-day interval before transfer into DD. The least squares regression line was used to estimate phase of this component on the day of transition into DD.

Statistical analyses were completed using JMP Software (SAS; Cary, NC) and CircStats Toolbox in Matlab for circular data^18^. Group means were compared using Student’s t-tests or ANOVA when variances were not unequal or Welch’s or Kruskal-Wallis tests when they were, followed by Wilcoxon pair-wise comparisons. The Rayleigh test was used to test for randomness of the distribution of projected activity onsets and Watson-Williams tests for differences in mean phase or phase angle^19^. The statistical significance level was set at p < 0.05 for all analyses unless otherwise specified.

## Results

### Experiment 1

#### Does bifurcation with DSI facilitate behavioral entrainment to T30?

##### Rhythm bifurcation in T24

**Figure 1:**
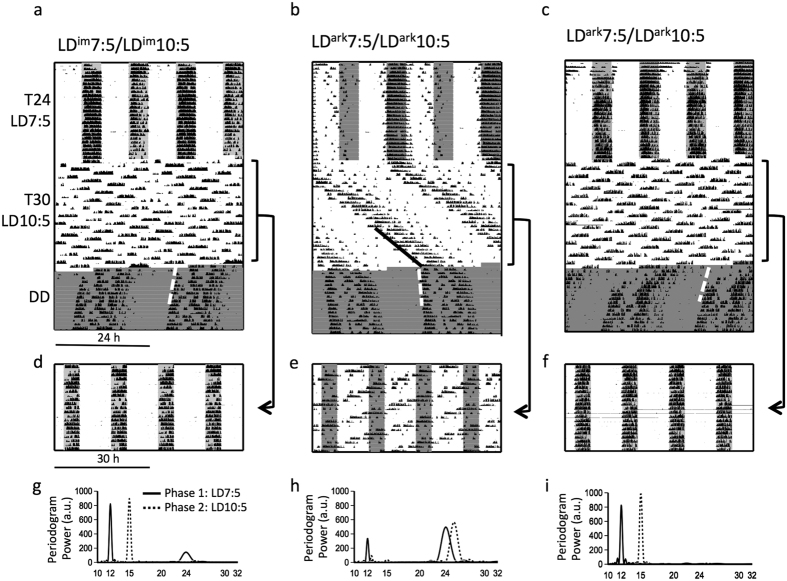
Representative double-plotted actograms of wheel-running activity in mice from Experiment #1 exposed to T24 LDLD, T30 LDLD and DD where the T24 and T30 phases included dim scotophase illumination (DSI) (**a**, LD^im^7:5/LD^im^10:5) or complete darkness (**b**, LD^ark^7:5/LD^ark^10:5). The actogram shown in C is not representative of the group, but illustrates anomalous bifurcation in dark nights (LD^ark^7:5/LD^ark^10:5) resembling the pattern shown in a. The same data from T30 are double-plotted modulo-24 h in (**a**–**c**) and again modulo-30 h in (**d–f**). Shading reflects times of relative darkness (dark shading is complete darkness; lighter shading is DSI). Shading in T30 LDLD is omitted in actograms plotted modulo-24 h (**a–c**) to aid in visualization of the underlying activity pattern, but shown for modulo-30 h plots (**d–f**). Dashed white lines illustrate best-fit lines to determine free-running period and projected activity onset phase in DD. In b, dark line shows best-fit line through free-running activity onsets in LDLD. Lomb-Scargle periodograms (**g,h**) for the final 14 complete LDLD cycles in T24 (Phase 1) and T30 (Phase 2) are shown for the same three animals shown above.

Figure 1a and b illustrate representative entrainment patterns of mice maintained under T24 and T30 LDLD cycles and subsequently exposed to DD. In Phase 1, LD^im^7:5 mice uniformly expressed a bifurcated pattern of entrainment characterized by robust wheel-running in each of the two separate scotophases and a near absence of activity during either of the intervening photophases (Fig. 1a, upper portion). Lomb-Scargle periodogram power was concentrated at 12 h with a much smaller peak at 24 h (Fig. 1g). In contrast, under LD^ark^7:5, mice typically failed to bifurcate: robust locomotor activity only occurred in one of the two daily scotophases with activity onsets generally anticipating lights out; activity in the second scotophase was sporadic and irregular in its timing (Fig. 1b, upper portion). Periodogram power showed peaks both at 12 h and at 24 h (Fig. 1h). Considered as a group, the LD^ark^7:5 mice entrained with significantly less symmetric distribution of activity between the scotophases (i.e. lower BSI) compared to LD^im^7:5 mice and with a greater fraction of activity occurring in the light (Table 1; see Supplementary Fig. S1). Periodogram power was significantly lower at 12 h and significantly higher at 24 h in LD^ark^7:5 versus LD^im^7:5 mice (Table 2). In 3 out of 20 mice exposed to LD^ark^7:5, however, the bifurcated pattern of entrainment could also be observed (Figs 1c; S1b).

##### Bifurcation in T30

After transfer from LD^im^7:5 to LD^im^10:5 in Phase 2 (Fig. 1a, middle portion), mice continued to confine wheel-running to the scotophases and divided it equally between them. Periodogram power shifted to 15 h and no secondary peak at 30 h was evident. In contrast, mice transferred from LD^ark^7:5 to LD^ark^10:5 (Fig. 1b, middle portion) commonly exhibited un-entrained activity that appeared to free-run across the photophases or show relative coordination. Apparent entrainment to LD^im^10:5 (Fig. 1d) but not to LD^ark^10:5 (Fig. 1e) is readily visualized in double-plots of the data at modulo 30 h. The existence of a free-running activity rhythm in LD^ark^10:5 is further evident in the pronounced periodogram peak around 25 h (Fig. 1h). Groupwise, LD^im^10:5 mice had significantly higher BSI values and significantly less activity in the light (Table 1). Periodogram power at 15 h was significantly greater for mice in LD^im^10:5 versus LD^ark^10:5 (Table 2). Periodogram power at 30 h was negligible (<7) in both groups, but was statistically greater in animals under LD^ark^10:5. More saliently, these mice with dark nights had significantly greater periodogram power in the free-running circadian range (23–26 h; Table 2). Mice with DSI had significantly higher EQ values than those with dark nights (Table 2). The anomalous mice that bifurcated in LD^ark^7:5 also adopted stable rhythms in LD^ark^10:5 (Fig. 1c).

##### Rhythmicity in DD

**Figure 2:**
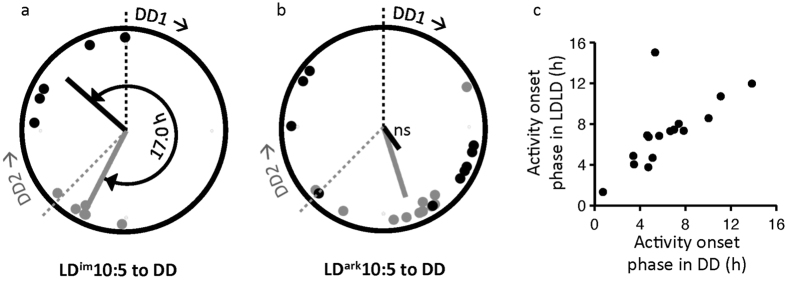
Circular plots of projected activity onsets in DD as a function of prior conditions ((**a**) LD^im^10:5, n = 10; (**b**) LD^ark^10:5, n = 20) and time of release into DD (DD1 = •; DD2 = •). Individual points (circles) and mean vectors (radial lines) are shown for each condition. Plots are in 24 h time beginning at DD1 (black dashed line) and proceeding clockwise. DD2 (shaded dashed lined) occurs 15 h after DD1. In panel c, the phase of the free-running component in LD^ark^10:5 (n = 16) strongly predicts the phase of the projected activity onset of the first cycle in DD. Values are plotted in h relative to 2000 PST. Correlation coefficient rises to 0.94 with removal of one outlier with mismatched phases (p < 0.000001).

Regardless of prior condition, within a few days of exposure to constant darkness (DD) in Phase 3, all animals exhibited typical free-running rhythms without discernible traces of prior bifurcation (Fig. 1a–c), a rigorously-characterized feature of bifurcated entrainment upon transfer to DD^9^. Free-running periods were significantly shorter in animals previously exposed to DSI (Table 1). For animals transferred from LD^im^10:5 to constant dark at DD_1_, projected activity onsets were significantly clustered (Rayleigh r = 0.86; p < 0.05) around a mean vector angle that anticipated DD_1_ onset by 3.2 h (Fig. 2a). The projected phases of animals transferred to DD after one additional LD^im^10:5 cycle (DD_2_) also were significantly clustered (r = 0.97; p < 0.01) around a mean vector angle 1.2 h before DD_2_. Comparing the two groups, the mean projected phases differed significantly from one another (Watson-Williams F_1,8_ = 33.3; p < 0.001). The phase angle difference relative to the respective DD onset conditions, however, did not vary between conditions (p > 0.1).

For mice transferred into constant conditions from LD^ark^10:5, projected activity onsets were only clustered after transfer at DD_2_ (r = 0.79; p < 0.001) with a mean value of 4.0 h before lights off, and were not clustered after transfer at DD_1_ (r = 0.29; p < 0.42; Fig. 2a). Projected phase after transfer to DD from LD^ark^10:5 was not significantly different for the mice released into DD_1_ versus DD_2_ (Watson-Williams test, F_1,18_ = 0.2; p = 0.68).

Prior to transfer to DD, a distinct non-entrained activity component could be visually identified in 16 of 20 animals in LD^ark^10:5 but none of the mice in LD^im^10:5. The phases of that non-entrained component were significantly clustered just prior to DD (r = 0.72; p < 0.001). Moreover, the projected phase of activity onset in DD was strongly correlated with the phase of the free-running activity onset prior to release into DD (r = 0.81; n = 16, p < 0.001; Fig. 2c).

### Experiment 2

#### Is DSI necessary for behavioral entrainment to T30 after bifurcation is induced?

##### Rhythm bifurcation in T24

Mice exposed to LD^im^8:4 followed by LD^im^7:5 exhibited the same bifurcated pattern of entrainment seen in Experiment #1 (see Supplementary Fig. S2) characterized by high values of BSI, low amounts of activity in the light (Table 1), and periodogram power concentrated at 12 h rather than at 24 h (Table 2).

##### Rhythm bifurcation in T30

In replication of Experiment #1, mice transferred from LD^im^7:5 to LD^im^10:5 continued to exhibit a bifurcated pattern of entrainment (representative actogram in Supplementary Fig. S2) as did those for whom the DSI was extinguished upon transfer to T30 (LD^im^7:5/LD^ark^10:5; Supplementary Fig. S2). Regardless of group, animals rarely exhibited visually obvious non-entrained activity components. In the example shown, the bifurcation was unstable prior to transfer to LD^ark^10:5. Quantitatively, there were no differences in BSI values in LD^im^10:5 versus LD^ark^10:5, although the latter group had significantly higher levels of activity in the light (Table 1). Periodogram power at 15 h was high for animals in both groups, while power at 30 h was negligible for both (<4; Table 2). Periodogram power in the circadian range was low (relative to that seen in Experiment #1) and did not differ between groups. Likewise, there was no group difference in EQ.

### Experiment 3

#### Is bifurcation per se necessary for behavioral entrainment to T30 or are DSI and short nights sufficient?

##### Entrainment under T24 and T30 LDLD cycles

**Figure 3:**
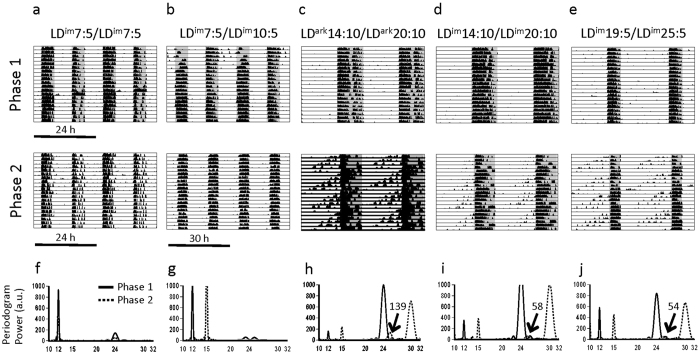
Representative actograms of subjects in Expt #3. Conventions as in Fig. 1. In panels h–j, the numerical value of the peak periodogram amplitude in the circadian range is indicated for Phase 2.

As in Experiments #1 and 2, the bifurcated pattern of entrainment was induced in LD^im^7:5 and maintained in LD^im^10:5 (Fig. 3a,b; Tables 1 and 2). BSI increased between Phase 1 and 2 for animals transferred to LD^im^10:5 (p < 0.001) but decreased with maintenance in LD^im^7:5 (p < 0.05; Group × Time interaction, p < 0.001).

##### Entrainment under T24 and T30 LD cycles

All mice exhibited stable entrainment to T24 LD cycles of Phase 1. Entrainment in LD^im^14:10 was characterized by lower activity levels in the light than in LD^ark^14:10 or LD^im^19:5 (Table 1; p < 0.001). As illustrated by representative actograms (Fig. 3c–e), in T30 nearly all LD mice exhibited visually obvious non-entrained activity components with periods between 23–26 h. Compared to mice in LD^im^10:5, each of the other T30 groups had lower EQs (Table 2; p < 0.01), more periodogram power in the 23–26 h and 30 h bands (Table 2; p < 0.05), and a greater portion of activity in the light (Table 1; p < 0.05). Comparing just the LD groups, the power of the 23–26 component was greater in LD^ark^20:10 than in LD^im^25:5, and power at 30 h was higher in LD^im^20:10 than in LD^im^25:5. The LD^im^20:10 group had lower activity in the light than did LD^ark^20:10 (p < 0.001; Wilcoxon rank sum). EQ did not differ significantly among the LD groups.

### Rhythmicity in DD

For no group did free-running period in DD differ between DD_1_ versus DD_2_ release, so data were collapsed across this variable. Free-running periods differed significantly by group. Periods were shorter in LD^im^10:5 than in LD^im^7:5, LD^ark^20:10, and LD^im^20:10 (p < 0.05; Table 1).

**Figure 4:**
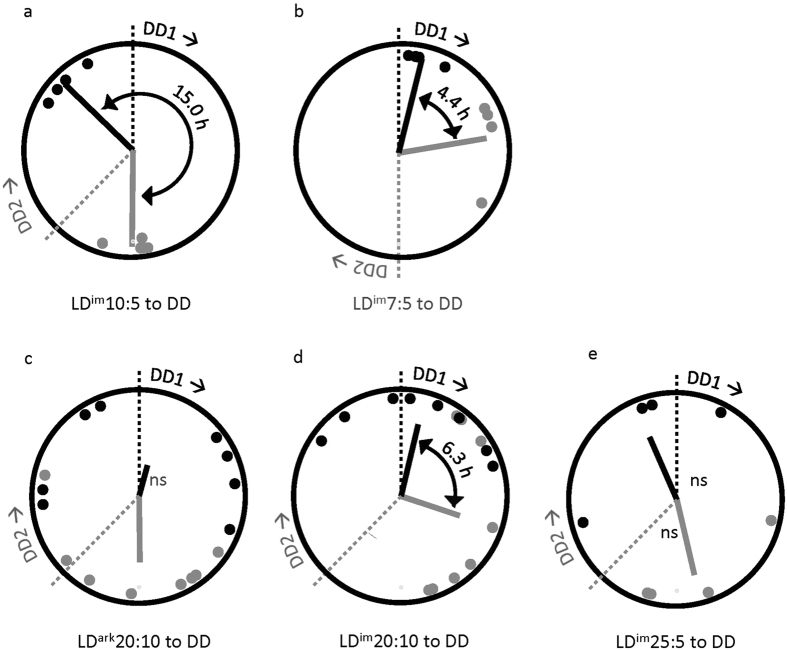
Circular plots of projected activity onsets in DD as a function of prior conditions ((**a**), LD^im^10:5, n = 8; (**b**) LD^im^7:5, n = 8; (**c**) LD^ark^20:10, n = 16; (**d**) LD^im^20:10, n = 16; and (**e**) LD^im^25:5, n = 8) and time of release into DD (DD1 = •; DD2 = •). Where there is no statistical evidence of clustered phases (ns), phase angles between vectors were not assessed. Conventions as in Fig. 2.

Upon release into constant conditions at DD_1_ and DD_2_, projected activity onsets of mice previously entrained to LD^im^10:5 were significantly clustered around mean vectors 3 h prior to the onset of their respective dark transitions (DD_1_: r = 0.92; p < 0.05; DD_2_: r = 0.99; p < 0.01; Fig. 4a) yielding mean phases 15.0 h apart (F_1,6_ = 189; p < 0.001). The phase angle of activity onset relative to the time of release into DD, however, did not differ by group (F_1,6_ = 0.1; p > 0.80). Bifurcated animals transferred to darkness from LD^im^7:5 were likewise significantly clustered (DD_1_: r = 0.98; p < 0.01; DD_2_: 0.98; p < 0.05; Fig. 4b). The mean vectors differed significantly from one another (F_1,6_ = 21.6; p < 0.01), and were also significantly different from a 12 h vector angle between groups (F_1,6_ = 59.2; p < 0.001).

In the cycle preceding release into DD, the phases of the free-running activity components were significantly clustered among LD^im^20:10 mice (r = 0.59; p < 0.01) but not LD^ark^20:10 (r = 0.21; p > 0.50) or LD^im^25:5 (r = 0.35; p > 0.30) animals. Following release of LD^ark^20:10 mice into DD, projected activity onsets were not significantly clustered after DD_1_ (r = 0.32; p > 0.40), but were significantly clustered around a mean vector 3.0 before DD_2_ (r = 0.68; p < 0.05; Fig. 4c). Time of release into DD significantly affected projected activity onset (F_1,6_ = 11.7; p < 0.01). Among LD^im^20:10 mice, there were significant clusterings of projected activity onsets around phase 0.9 h after DD_1_ (r = 0.75; p < 0.01) and 7.2 h before DD_2_ (r = 0.64; p < 0.05), respectively (Fig. 4d). Time of release into DD again significantly affected projected activity onset. After LD^im^25:5, onsets appear clustered after neither DD_1_ (r = 0.37; p > 0.60) nor DD_2_ (r = 0.55; p > 0.30; Fig. 4e), but we can conclude little given the small sample size. Time of release into DD did not affect final distribution of onsets (F_1,6_ = 1.7; p > 0.20).

### Predictability of EQ T30

**Figure 5:**
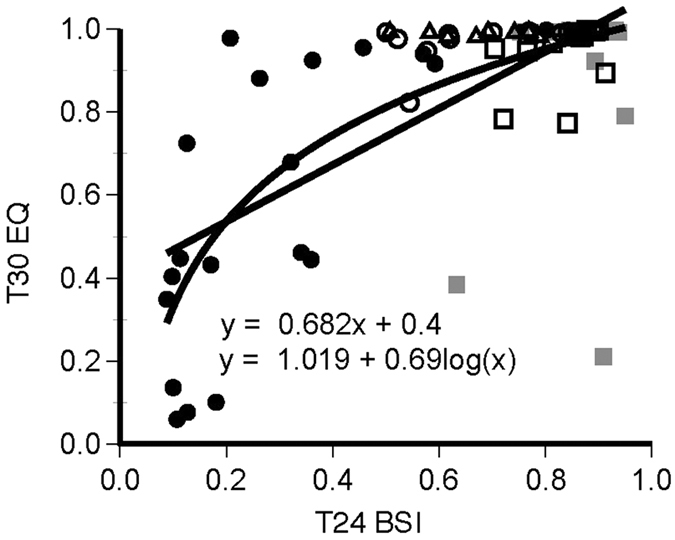
Relationship between BSI in final 14 cycles of Phase 1 (LDLD7:5:7:5) and EQ in final 14 cycles of Phase 2 (LDLD10:5:10:5) for all three experiments (n = 69). Open and black filled symbols are groups exposed to DSI or dark throughout. Solid grey are animals moved from DSI to dark. Circles, squares and triangles indicate data come from experiments 1–3, respectively. Solid black lines indicate best fitting linear and logarithmic functions.

Collapsed across all groups exposed to LDLD cycles in all three experiments, BSI values in Phase 1 were significantly correlated with EQs in T30 (linear regression adjusted R^2^ = 0.45, p < 0.001; log linear regression adjusted R^2^ = 0.56, p < 0.001; Fig. 5). Comparable correlation coefficients were obtained in LD^ark^7:5/LD^ark^10:5 (R^2^ = 0.48 and R^2^ = 0.54) but values were uncorrelated in LD^im^7:5/LD^im^10:5 or LD^im^7:5/LD^ark^10:5 animals where there was a limited range of variance.

## Discussion

The addition of very dim light at night categorically altered the manner in which mice entrained to 24 h LDLD cycles. With conventionally dark nights, wheel-running rhythms entrained as expected from decades of work with “skeleton” photoperiods, the most commonly studied LDLD cycle in the circadian literature: activity is decidedly concentrated in one of the two scotophases, and the remaining light:dark:light portion of the cycle is an interval of general inactivity except for some wheel-running just before and/or after the preferred scotophase. With dim night-time illumination, however, the entrainment pattern is bifurcated such that robust locomotor activity occurs in each of the two daily scotophases and not during intervening light phases. By evaluating how evenly activity is distributed between any two adjacent scotophases, the BSI provides an objective measure of the waveform differences in dim versus dark nights. The intensity of nighttime illumination producing this effect approximates that of starlight or dim moonlight and thus, aside from its spectral qualities, reflects conditions that could be experienced in the field, although the LDLD condition is completely artificial. The results extend prior findings in Syrian and Siberian hamsters in which bifurcation and its induction under dim light have been extensively characterized^2^,^5^,^7^,^9^,^10^,^20^.

Breaking new ground, dim nighttime illumination markedly altered the response to light cycles generally considered beyond the range of entrainment. The expected pattern of *failed* entrainment to T30 LDLD was observed in mice that never experienced dim nighttime illumination: nearly all exhibited visually obvious free-running rhythms that were objectively reflected in lower EQ values. By assessing the relative strength of periodogram power matching the lighting cycle in T15 and/or 30 versus that of the free-running circadian clock (~23–26), the EQ captures objectively the degree to which the animal’s activity is environmentally controlled. In mice with a history of dim light exposure, by contrast, activity rhythms appeared closely aligned with the light cycle; there was rarely any hint of free-running activity components; and EQ values approached unity. But masking of activity by LD cycles could also yield substantial periodogram power at a period matching T, and visually obvious free-running components may have relatively small signatures in the periodogram. Thus, a corroboratory entrainment criterion, phase control, was evaluated under constant conditions.

In both Experiments 1 and 3, the phase of the rejoined rhythm emerging in DD after LD^im^10:5 was tightly clustered and strictly controlled by the time of release in DD. In contrast, free-running phases of rhythms in DD after LD^ark^10:5 were either not clustered or phase was strongly correlated with the phase of the prior identifiable free-running component (Fig. 2). Thus, dim light afforded control of both period and phase in T15/T30 cycles. While the activity rhythm reverts to unimodality in DD, the phase of the onsets is completely predicted by the time of release into DD, in marked contrast to the LD^ark^10:5 group and LD groups. This reversion to unimodality is to be expected as prior experiments from multiple species demonstrate that, while bifurcation is a bona fide entrainment state, it does not persist in the absence of an LDLD cycle^9^.

Because mice bifurcated under dim light in T24 LDLD adapted equally well to T30 regardless of whether or not the dim light was continued (Expt 2), and because entrainment to LD^ark^10:5 appears to depend profoundly on history (Expt 1 vs 2), this extraordinary behavioral entrainment cannot be attributed to an acute masking response to T30 conditions. This result does not, however, resolve whether superior entrainment derives principally from rhythm bifurcation that happened to be induced by dim light or from a long-lasting after-effect of dim exposure independent of any effect on bifurcation. Arguing for the former, mice that had bifurcated rhythms in LD^ark^7:5 entrained to T30 LDLD in a comparable fashion as dim-exposed mice, although the low incidence of bifurcation in dark nights precluded rigorous statistical evaluation of this possibility. The strong correlation between BSI in LD7:5 conditions and EQ in T30 also supports this conclusion. Conversely, in the absence of bifurcation, no other dimension of the light stimulus allowed for comparable entrainment. Specifically, exposure to LD^im^20:10 and LD^im^25:5 did attenuate but not eliminate visually obvious free-running components or fully suppress periodogram power in the 23–26 h range suggesting that neither dim light nor a short scotophase was sufficient for entrainment to T30. Moreover, the categorical effect on entrainment of dim light in T24 and T30 LDLD did not generalize to LD conditions where only quite modest effects on percent of activity in the light were noted. We did not explicitly test whether dim light, in the absence of prior bifurcation, might also facilitate entrainment to LD 10:5.

Although the 24 h LDLD stimulus was equivalent to a 12 h LD cycle, bifurcated mice entrained to these conditions with 24 rather than 12 h rhythmicity. If the entrained rhythm had been 12 h, then activity occurring in the odd numbered scotophases would be wholly equivalent to that in the even numbered scotophases. On the contrary, distinct activity patterns were clearly visible between the alternate scotophases (e.g., Fig. 1b) and were reflected in substantial 24 h power in the Lomb-Scargle periodogram. (Activity patterns that were identical in successive scotophases would fall in opposite phases of a 24 h and thus yield no power in this cosine-fitting model). Additionally, instigation of DD at the beginning of the dark period that was the original night resulted in a joined rhythm with a phase nearly coincident with the timing of release into DD. Again, were the pacemaker oscillating with a 12 h period, initiation of DD 12 h later (one additional LD7:5 cycle) would be expected to yield a re-joined rhythm phased 12 h later. Instead, DD beginning during the afternoon scotophase resulted in only a 4 h delay in phase compared to DD beginning in the nighttime scotophase. Analogous experiments in hamsters yielded very similar results^9^ and were interpreted in terms of dynamic influences of oscillators underlying the bifurcated pattern of entrainment.

Given the asymmetric entrainment to alternate scotophases in T24, it was somewhat surprising to observe in the dim-exposed T30 mice of Experiment #1 that bouts of activity appeared much more evenly entrained and that values of BSI were higher than in T24. In the context of EQ values near 1, this metric indicates stable and more even division between activity components. Although BSI of dark-exposed mice also increased in T30, in the context of low EQ values, this reflects a more frequent occurrence of divided scotophase activity as the rhythm free-runs relative to the lighting cycle. Since mice chronically maintained in *LD*^*im*^*7:5* in Experiment #3 exhibited a decrease in BSI over time, the increase in BSI in T30 was photoperiod-specific. Thus, activity rhythms are most accurately described as 24 h in T24 LDLD but 15 h in T30 LDLD, a characterization that is consistent with the steady state phase of rhythms after transfer to DD.

An interpretation that the circadian system is classically or authentically entrained to a 15 h period (or even a 30 h period), however, is complicated by consideration of other conventional criteria. First, in many previous studies, mice that entrained to T cycles shorter or longer than 24 h reliably exhibited period after-effects in DD^21^,^22^. While some significant differences in tau were noted between groups, T30 LDLD in the present experiments never yielded particularly long or short periods as might be expected for 30 h or 15 h entrainment, respectively. Moreover, LDLD entrainment in Experiment #3 yielded no period after-effect in T24 versus T30. Second, entrainment theory predicts and several studies confirm that, relative to dark onset, activity onsets will be advanced and delayed when T is longer or shorter than 24 h, respectively. In marked contrast, activity onsets in T15/30 were reliably close to dark onset. In an analogous experiment of bifurcated Syrian hamsters entrained to gradually lengthening or shortening T cycles, phase angles were modified as predicted only in a narrow range (±2 h) around 24 h. Beyond this point, they adopted a comparable fixed relationship to light offset (manuscript in preparation). Thus, we surmise that activity rhythms under T15/30 are controlled by different mechanisms than those operating near 24 h. Under T15/30 conditions, endogenous circadian influence on activity may be greatly attenuated such that rhythms may be controlled directly by driving effects of the light/dark cycle. This account differs from traditional masking accounts insofar as here we observed no evidence for a persistent free-running circadian rhythm in LD^im^10:5 that continues to cycle but only temporarily loses control over overt activity in the presence of the light cycle. In the majority of LD^im^10:5 animals, we were unable to observe any free-running component in T15/30 either by eyefit or by periodogram power (Table 2). Further, the total amount of dim light received by the LD^im^10:5 group per cycle is the same as that received by the LD^im^20:10 group. Yet, we were able to discern a free-running component with ease in the LD^im^20:10 group, and periodogram power in that range more than double for that group (Table 2). Thus, we argue that LD^im^10:5 results in a largely-attenuated and acutely-driven behavioral rhythm, and that robust circadian control re-emerges only after the LDLD cycle is discontinued and at a phase established by the transition to constant darkness.

Under conditions of dampened oscillations, it may be argued whether control of behavior by the lighting cycle should be labeled circadian “entrainment”, which is typically studied under conditions of robust endogenous rhythmicity. By the same token, control of activity by “masking” would require a robust endogenous oscillator mechanism from which an overt rhythm has been decoupled. Outside the context of bifurcation, DSI has a host of effects on classical entrainment and re-entrainment paradigms in other species^1^,^2^,^3^,^4^,^23^. And likewise, bifurcation in T24 meets multiple entrainment criteria^5^,^8^,^24^. Thus, for their joint effects in T30, we cautiously retain the usage of “behavioral entrainment” acknowledging that further characterization of this novel rhythmicity is required.

While the mechanisms for the extraordinary behavioral results observed here are unknown, we speculate that the anti-phasic gene expression in the core and shell of SCN neurons found under bifurcation^6^,^7^ reflects a reorganization of oscillators within the SCN that facilitates behavioral adjustment to challenging light-dark schedules. An and colleagues demonstrated enhanced phase-resetting following pharmacological and genetic manipulations that dampened and dissociated rhythmicity of SCN networks^25^. There is other evidence, however sparse, that the relationship between the SCN and the LD cycle can be dramatically altered by short-term lighting manipulations, such as constant light or specific phase-shifting protocols^26^,^27^. While we may speculate that some of these underlying mechanisms could be shared, we cannot rule out the possibility of dim light and/or bifurcation history effects on extra-SCN physiology, including the intergeniculate leaflet (IGL)^23^ or retina. Further experiments are necessary to definitively determine the underlying mechanisms of the behavioral patterns observed here.

## Additional Information

**How to cite this article**: Harrison, E. M. *et al*. Extraordinary behavioral entrainment following circadian rhythm bifurcation in mice. *Sci. Rep.* **6**, 38479; doi: 10.1038/srep38479 (2016).

**Publisher’s note:** Springer Nature remains neutral with regard to jurisdictional claims in published maps and institutional affiliations.

## Supplementary Material

Supplementary Information

## Figures and Tables

**Table 1:** Wheel-running activity distribution and free-running periods in T24, T30 and DD.

	Condition	Bifurcation Symmetry Index (BSI)	Activity in Light (%)		Condition	Bifurcation Symmetry Index (BSI)	Activity in Light (%)		Free-running Period (h)	N
**Expt 1**	**LDim7:5**	0.65 ± 0.04	2.0 ± 0.5	**→**	**LDim10:5**	0.79 ± 0.03	2.0 ± 2.4	**→**	23.65 ± 0.07	10
	**LDark7:5**	0.31 ± 0.05	16.6 ± 2.1	**→**	**LDark10:5**	0.48 ± 0.05	27.5 ± 3.4	**→**	23.92 ± 0.05	20
		p < 0.001	p < 0.0001*			p < 0.001*	p < 0.0001*		p < 0.01	
**Expt 2**	**LDim7:5**	0.84 ± 0.02	1.6 ± 0.7	**→**	**LDim10:5**	0.78 ± 0.05	2.6 ± 0.6	**→**	n/d	15
	**LDim7:5**	0.87 ± 0.02	1.8 ± 0.4	**→**	**LDark10:5**	0.79 ± 0.04	9.3 ± 2.7	**→**	n/d	16
		ns	ns			ns	p < 0.05*			
**Expt 3**	**LDim7:5**	0.70 ± 0.04	0.7 ± 0.1	**→**	**LDim7:5**	0.60 ± 0.03	1.4 ± 0.3a	**→**	23.78 ± 0.04d	8
	**LDim7:5**	0.67 ± 0.03	2.5 ± 2.0	**→**	**LDim10:5**	0.85 ± 0.02	2.2 ± 1.1a	**→**	23.65 ± 0.02	8
		ns	ns			p < 0.001	ns			
	**LDark14:10**	n/d	1.7 ± 0.2b	**→**	**LDark20:10**	n/d	20.2 ± 3.7c	**→**	23.82 ± 0.04d	16
	**LDim14:10**	n/d	0.8 ± 0.2	**→**	**LDim20:10**	n/d	5.3 ± 1.1	**→**	23.75 ± 0.03d	16
	**LDim19:5**	n/d	3.5 ± 1.4b	**→**	**LDim25:5**	n/d	10.5 ± 2.9	**→**	23.56 ± 0.14	8
			p < 0.001*				p < 0.01*		p < 0.01*	

^^^One group, LDim7:5/LDim7:5, remains in T24 LD in Phase 2.

^*^Welch’s or Kruskal-Wallis test not assuming equal variances.

Letters indicate significantly different values from all T30 groups (a); from LDim14:10 (b); or from LDim20:10 (c) and from LD10:5 (d). n/d: not determined.

**Table 2:** Lomb-Scargle periodogram analysis of wheel-running entrainment in T24 and T30.

	Phase 1 (T24)				Phase 2 (T30)				
	Condition	Lomb-Scargle Periodogram Power			Condition	Lomb-Scargle Periodogram Power			EQ
		12 h	24 h			15 h	30 h	23–26 h	
**Expt 1**	**LDim7:5**	818.2 ± 57.0	94.2 ± 19.3	**→**	**LDim10:5**	913.6 ± 83.7	2.3 ± 1.3	24.4 ± 11.3	0.97 ± 0.02
	**LDark7:5**	446.4 ± 43.8	330.3 ± 45.9	**→**	**LDark10:5**	503.9 ± 83.3	6.0 ± 1.0	266.4 ± 49.0	0.59 ± 0.08
		p < 0.001	p < 0.001*			p < 0.01	p < 0.05	p < 0.001*	p < 0.001*
**Expt 2**	**LDim7:5**	859.9 ± 33.1	24.1 ± 7.6	**→**	**LDim10:5**	969.5 ± 59.8	1.0 ± 0.4	47.8 ± 18.6	0.95 ± 0.02
	**LDim7:5**	855.8 ± 39.8	10.4 ± 4.1	**→**	**LDark10:5**	878.2 ± 91.2	2.7 ± 1.0	81.3 ± 44.2	0.89 ± 0.06
		ns	ns*			ns*	ns	ns*	ns*
**Expt 3**	**LDim7:5**	538.4 ± 59.2	59.9 ± 14.7	**→**	**LDim7:5**	473.0 ± 83.3^	36.5 ± 10.4^	n/d	n/d
	**LDim7:5**	638.9 ± 63.0	86.1 ± 23.5	**→**	**LDim10:5**	744.9 ± 88.0	1.6 ± 0.6a	7.6 ± 0.9a	0.99 ± .002a
	**LDark14:10**	n/d	n/d	**→**	**LDark20:10**	n/d	577.4 ± 70.3	71.0 ± 19.1b	0.83 ± 0.06
	**LDim14:10**	n/d	n/d	**→**	**LDim20:10**	n/d	685.6 ± 48.4b	37.5 ± 5.8	0.94 ± 0.01
	**LDim19:5**	n/d	n/d	**→**	**LDim25:5**	n/d	497.1 ± 20.5	19.5 ± 4.2	0.96 ± 0.01
							p < 0.001*	p < 0.001*	p < 0.001*

^^^One group, LDim7:5/LDim7:5, remains in T24 LD in Phase 2 (assessed at 12 and 24).

^*^Welch’s or Kruskal-Wallis test not assuming equal variances.

a: differs significantly from all other groups. b: differs significantly from LDim25:5. n/d: not determined.

## References

[b1] Evans J. A., Elliott J. A. & Gorman M. R. Dim nighttime illumination accelerates adjustment to timezone travel in an animal model. Curr. Biol. 19, R156–7 (2009). 19243688 10.1016/j.cub.2009.01.023

[b2] Frank D. W., Evans J. A. & Gorman M. R. Time-dependent effects of dim light at night on re-entrainment and masking of hamster activity. J. Biol. Rhythms 25, 103–112 (2010). 20348461 10.1177/0748730409360890

[b3] Gorman M. R., Kendall M. & Elliott J. A. Scotopic illumination enhances entrainment of circadian rhythms to lengthening light:dark cycles. J. Biol. Rhythms 20, 38–48 (2005). 15654069 10.1177/0748730404271573

[b4] Evans J. A., Elliott J. A. & Gorman M. R. Circadian effects of light no brighter than moonlight. J. Biol. Rhythms 22, 356–67 (2007). 17660452 10.1177/0748730407301988

[b5] Gorman M. R. & Elliott J. A. Entrainment of 2 subjective nights by daily light:dark:light:dark cycles in 3 rodent species. J. Biol. Rhythms 18, 502–12 (2003). 14667151 10.1177/0748730403260219

[b6] Watanabe T. . Bimodal clock gene expression in mouse suprachiasmatic nucleus and peripheral tissues under a 7-hour light and 5-hour dark schedule. J. Biol. Rhythms 22, 58–68 (2007). 17229925 10.1177/0748730406295435

[b7] Yan L., Silver R. & Gorman M. Reorganization of suprachiasmatic nucleus networks under 24-h LDLD conditions. J. Biol. Rhythms 25, 19–27 (2010). 20075297 10.1177/0748730409352054 PMC3275439

[b8] Raiewski E. E., Elliott J. A., Evans J. A., Glickman G. L. & Gorman M. R. Twice daily melatonin peaks in Siberian but not Syrian hamsters under 24 h light:dark:light:dark cycles. Chronobiol. Int. 1–10, doi: 10.3109/07420528.2012.719965 (2012). PMC4917013 23003567

[b9] Evans J. A., Elliott J. A. & Gorman M. R. Dynamic interactions between coupled oscillators within the hamster circadian pacemaker. Behav Neurosci 124, 87–96 (2010). 20141283 10.1037/a0018088 PMC2830911

[b10] Gorman M. R. & Steele N. A. Phase angle difference alters coupling relations of functionally distinct circadian oscillators revealed by rhythm splitting. J. Biol. Rhythm. 21, 195–205 (2006). 10.1177/0748730406287665 16731659

[b11] Goldman B. D. & Elliott J. A. Photoperidosm and seasonality in hamsters: the role of the pineal gland in Processing of environmental information in vertebrates: Proceedings in Life Sciences (ed. Stetson M. H.) 203–218 (1988).

[b12] Evans J. A., Elliott J. A. & Gorman M. R. Photoperiod differentially modulates photic and nonphotic phase response curves of hamsters. Am. J. Physiol. Regul. Integr. Comp. Physiol. 286, R539–46 (2004). 14644756 10.1152/ajpregu.00456.2003

[b13] Glickman G. L. . Photic sensitivity for circadian response to light varies with photoperiod. J. Biol. Rhythms 207, 308–317 (2012). 10.1177/0748730412450826 22855575

[b14] Glickman G. L., Harrison E. M., Elliott J. A. & Gorman M. R. Increased photic sensitivity for phease resetting but not melatonin suppression in Siberian hamsters under short photoperiods. Horm. Behav. 65, 301–307 (2014). 24440383 10.1016/j.yhbeh.2014.01.002 PMC3963461

[b15] Harrison E. M. & Gorman M. R. Rapid adjustment of circadian clocks to simulated travel to time zones across the globe. J. Biol. Rhythms, doi: 10.1177/0748730415598875 (2015). 26275871

[b16] Ruf T. The Lomb-Scargle Periodogram in Biological Rhythm Research: Analysis of Incomplete and Unequally Spaced Time-Series. Biol. Rhythm Res. 30, 178–201 (1999).

[b17] Refinetti R., Lissen G. C. & Halberg F. Procedures for numerical analysis of circadian rhythms. Biol. Rhythm Res. 38, 275–325 (2013). 10.1080/09291010600903692 PMC3663600 23710111

[b18] Berens P. CircStat: A MATLAB toolbox for circular statistics. J. Stat. Softw. 31, 1–21 (2009).

[b19] Batschelet E. Circular Statistics in Biology. (Academic Press, 1981).

[b20] Gorman M. R., Elliott J. A. & Evans J. A. Plasticity of hamster circadian entrainment patterns depends on light intensity. Chronobiol. Int. 20, 233–248 (2003). 12723883 10.1081/cbi-120018576

[b21] Molyneux P. C., Dahlgren M. K. & Harrington M. E. Circadian entrainment aftereffects in suprachiasmatic nuclei and peripheral tissues *in vitro*. Brain Res. 8, 127–134 (2008). 10.1016/j.brainres.2008.05.091 18598681

[b22] Aton S. J., Block G. D., Tei H., Yamazaki S. & Herzog E. D. Plasticity of circadian behavior and the suprachiasmatic nucleus following exposure to non-24-hour light cycles. J. Biol. Rhythms 19, 198–207 (2004). 15155006 10.1177/0748730404264156

[b23] Evans J. A., Carter S. N., Freeman D. A. & Gorman M. R. Dim nighttime illumination alters photoperiodic responses of hamsters through the intergeniculate leaflet and other photic pathways. Neuroscience 202, 300–308 (2012). 22155265 10.1016/j.neuroscience.2011.11.037 PMC3578228

[b24] Harrison E. M. & Gorman M. R. Changing the waveform of circadian rhythms: considerations for shift-work. Front. Neurol. 3, 1–7 (2012). 22557994 10.3389/fneur.2012.00072 PMC3340571

[b25] An S. . A neuropeptide speeds circadian entrainment by reducing intercellular synchrony. Proc. Natl. Acad. Sci. USA 110, E4355–61 (2013). 24167276 10.1073/pnas.1307088110 PMC3832006

[b26] Knoch M. E. . Short-term exposure to constant light promotes strong circadian phase-resetting responses to nonphotic stimuli in Syrian hamsters. Eur. J. Neurosci. 19, 2779–2790 (2004). 15147311 10.1111/j.0953-816X.2004.03371.x

[b27] Ruby N. F., Saran a, Kang T., Franken P. & Heller H. C. Siberian hamsters free run or become arrhythmic after a phase delay of the photocycle. Am. J. Physiol. 271, R881–90 (1996). 8897977 10.1152/ajpregu.1996.271.4.R881

