# Supplementary material for: Extraordinary behavioral entrainment following circadian rhythm bifurcation in mice
Source: Sci Rep. 2016 Dec 8;6:38479. doi: 10.1038/srep38479 (PMC5144065; doi:10.1038/srep38479)
Supplement: Supplementary Information [file srep38479-s1.pdf]

Extraordinary behavioral entrainment following circadian rhythm bifurcation in mice

Harrison, Walbeek, Sun, Poonawala, Johnson & Gorman

Supplementary Online Material

2 pages

Supplementary Figure S1

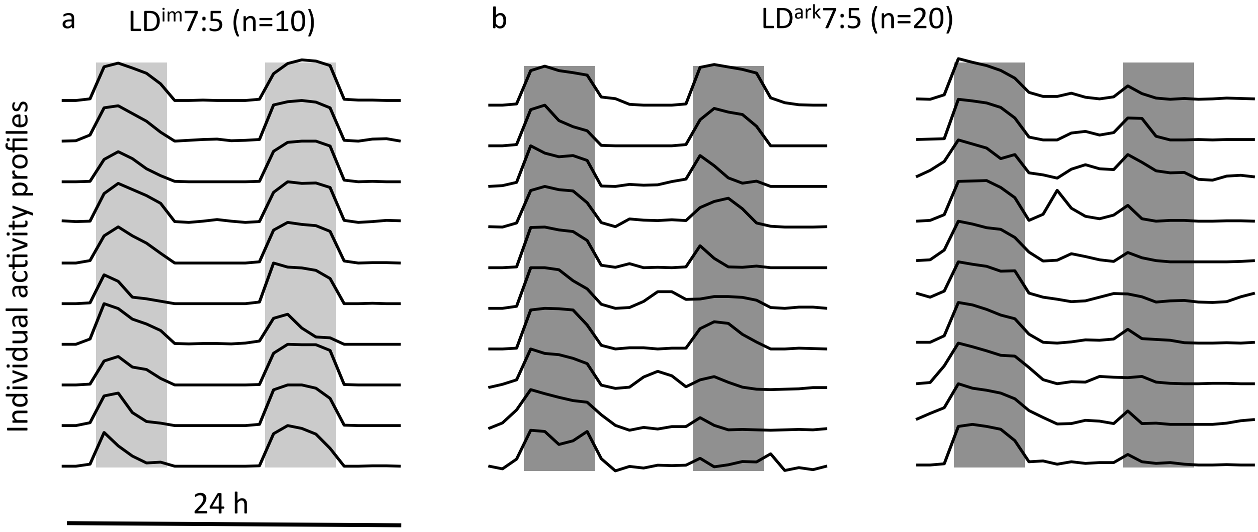

Supplementary Figure S1. Single-plotted 14-day activity profiles for all subjects in Expt. #1. Times of dim and dark scotophases are indicated by lighter and darker shading, respectively. Plots are normalized to a constant area under the curve. Profiles are ordered with respect to Bifurcation Symmetry Index (decreasing from top to bottom within each group).

Supplementary Figure S2

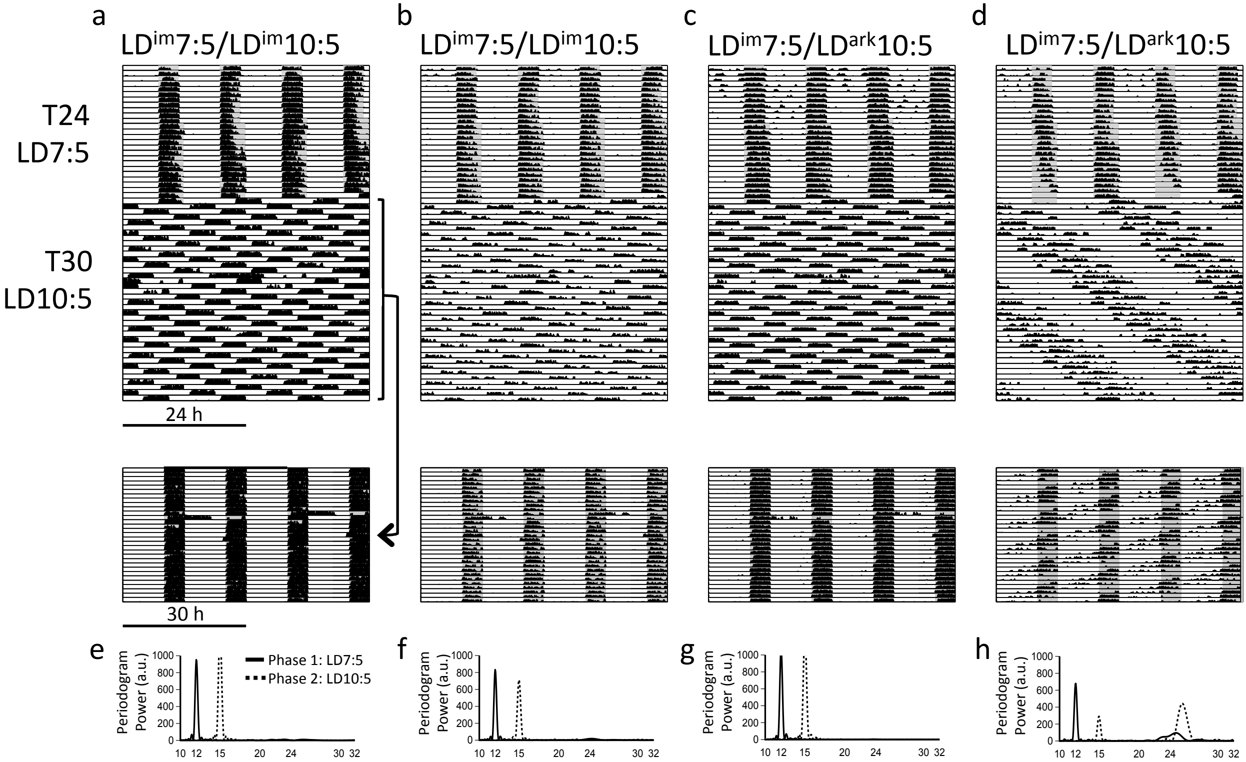

Supplementary Figure S2. Representative actograms (2 per group) of subjects in Expt # 2. Conventions as in Figure 1. Shading in T30 LDLD is omitted in actograms plotted modulo-24 h to aid in visualization of the underlying activity pattern, but shown for modulo-30 h plots. Midway through T30 LDLD, a mechanical failure occurred causing mice to be exposed to darkness for ~10 h instead of 5 h for one cycle.
